# Hepatotoxicity of Pyrrolizidine Alkaloid Compound Intermedine: Comparison with Other Pyrrolizidine Alkaloids and Its Toxicological Mechanism

**DOI:** 10.3390/toxins13120849

**Published:** 2021-11-28

**Authors:** Ziqi Wang, Haolei Han, Chen Wang, Qinqin Zheng, Hongping Chen, Xiangchun Zhang, Ruyan Hou

**Affiliations:** 1 State Key Laboratory of Tea Plant Biology and Utilization, School of Tea and Food Science & Technology, Anhui Agricultural University, Hefei 230036, China; wangziqi199710@163.com; 2 Tea Research Institute, Chinese Academy of Agricultural Sciences, Hangzhou 310008, China; hanhaolei@tricaas.com (H.H.); wangchen@tricaas.com (C.W.); zhengqinqin@tricaas.com (Q.Z.); thean27@tricaas.com (H.C.); 3 Graduate School, Chinese Academy of Agricultural Sciences, Beijing 100081, China; 4 Key Laboratory of Tea Quality and Safety & Risk Assessment, Ministry of Agriculture, Hangzhou 310008, China

**Keywords:** pyrrolizidine alkaloids, intermedine, liver injury, cytotoxicity, toxicity mechanism

## Abstract

Pyrrolizidine alkaloids (PAs) are common secondary plant compounds with hepatotoxicity. The consumption of herbal medicines and herbal teas containing PAs is one of the main causes of hepatic sinusoidal obstruction syndrome (HSOS), a potentially life-threatening condition. The present study aimed to reveal the mechanism underlying the cytotoxicity of intermedine (Im), the main PA in *Comfrey*. We evaluated the toxicity of the retronecine-type PAs with different structures to cell lines derived from mammalian tissues, including primary mouse hepatocytes, human hepatocytes (HepD), mouse hepatoma-22 (H_22_) and human hepatocellular carcinoma (HepG2) cells. The cytotoxicity of Im to hepatocyte was evaluated by using cell counting kit-8 assay, colony formation experiment, wound healing assay and dead/live fluorescence imaging. In vitro characterization showed that these PAs were cytotoxic and induced cell apoptosis in a dose-dependent manner. We also demonstrated that Im induced cell apoptosis by generating excessive reactive oxygen species (ROS), changing the mitochondrial membrane potential and releasing cytochrome c (Cyt c) before activating the caspase-3 pathway. Importantly, we directly observed the destruction of the cell mitochondrial structure after Im treatment through transmission electron microscopy (TEM). This study provided the first direct evidence of Im inducing hepatotoxicity through mitochondria-mediated apoptosis. These results supplemented the basic toxicity data of PAs and facilitated the comprehensive and systematic evaluation of the toxicity caused by PA compounds.

## 1. Introduction

Herbal medicines and plant-related products are indispensable for human life in many countries. However, hepatic sinusoidal obstruction syndrome (HSOS) has been induced after ingestion of herbals and plant-related products in many countries and has attracted global attention [1,2]. HSOS is clinically characterized by hepatic congestion and swelling, blockage of liver blood vessels, detachment of sinusoidal endothelial cells and hepatic dysfunction [2,3]. Through an in-depth study of these HSOS patients, it was found that most of them were exposed to pyrrolizidine alkaloids (PAs) through the consumption of herbals and herbal teas [2,4]. Despite the great advances in medical technology in the past few decades, there is still no effective clinical therapy for PA-induced HSOS cases. Therefore, extensive and in-depth research on PA-induced hepatotoxicity and the development of effective treatments is urgently needed.

PAs are common plant secondary metabolites, which are mainly distributed in these three families: *Boraginaceae*, *Asteraceae* and *Fabaceae* [5]. To date, more than 660 PAs and PA N-oxides have been identified in about 3% of flowering plants [5,6]. PAs are potential carcinogens that directly enter the human body through the food chain (such as grains, meat, milk, eggs and honey) and poison livestock that graze on PA-contaminated plants [7,8,9,10]. In the previous study, PAs/PA N-oxides were detected in green tea, black tea, dark tea and chrysanthemum [11]. The hepatotoxicity, carcinogenicity, pulmonary toxicity, genotoxicity and heredity of PAs in humans has attracted a great deal of attention from toxicologists [4,8,12,13,14].

As part of PAs, PA N-oxides caused hepatotoxicity through the biotransformation to the corresponding PAs [15]. As the largest detoxification organ in mammals, the liver is the main target organ for PAs and PA N-oxides. Therefore, these alkaloids have the most significant hepatotoxicity [1,8,16,17]. Given the severe hepatotoxicity of PAs and following a comprehensive risk assessment of this hepatocarcinogen, many countries and international organizations, such as the European Union, the Germany Federal Institute for Risk Assessment, the Australia New Zealand Food Authority (ANZFA), the United Kingdom and the Netherlands, have established intake limits or recommended daily intakes of PAs [18,19,20].

**Figure 1:**
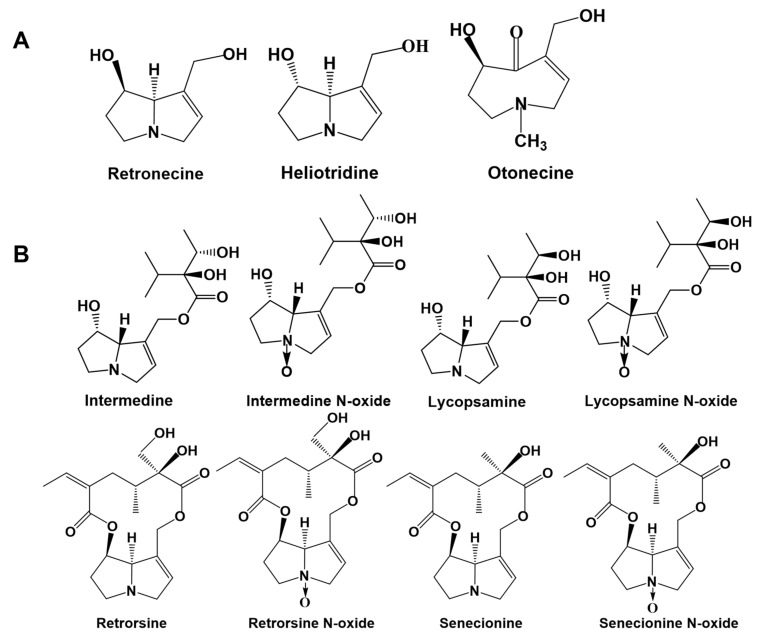
Structures of 1,2-unsaturated pyrrolizidine alkaloids (PAs). (**A**) Typical structures of retronecine-type, heliotridine-type and otonecine-type. (**B**) Structures of intermedine (Im), intermedine N-oxide (ImNO), lycopsamine (La), lycopsamine N-oxide (LaNO), retrorsine (Re), retrorsine N-oxide (ReNO), senecionine (Sc) and senecionine N-oxide (ScNO).

PAs consist of saturated PAs and unsaturated PAs. The unsaturated PAs are toxic because they contain 1, 2-unsaturated double bonds and are mainly divided into three types: retronecine-type, heliotridine-type and otonecine-type (Figure 1A) [18,21]. The saturated PAs are generally non-toxic, so the unsaturated PAs have become a hot spot because of their toxicity and pathogenicity. Many studies have focused on PAs with higher hepatotoxicity, such as retronecine-type PAs [13,22,23,24], while few studies focus on PAs with low or medium toxicity. To fully understand the hazards of PAs in plants and food, and systematically and comprehensively evaluate the toxicity of PAs, it is necessary to obtain basic toxicological data and toxicity mechanisms of PA compounds with high exposure levels and low or medium toxicity.

Previous studies have demonstrated that the toxicity of PAs was related to their structural characteristics [21,25,26,27]. This study showed that senecionine (retronecine-type), echimidine (retronecine-type), heliotrine (heliotridine-type) and senkirkine (otonecine-type) were toxic to HepaRG cells. The cytotoxicity of these four PAs was as follows: senecionine > echimidine/heliotrine > senkirkine [21]. It was thus extrapolated that, among the three 1,2-unsaturated PAs, the retronecine-type was the most toxic PA. PAs were further classified as diesters and monoesters, based on the number of ester groups on the branches in the retronecine structure. Afterwards, it was found that, among the retronecine-type PAs, the largest number of pyrrole-protein adducts was produced by open-ring diesters, followed by the macrocyclic diesters, while the smallest number was produced by monoesters, and the production of pyrrole-protein adducts was the main cause of HSOS [28,29]. The order of toxicity of retronecine-type PAs was demonstrated as follows: open-ring diester > macrocyclic diester > monoester [25,30,31]. Monoesters appeared to have the lowest toxicity, so this was easily overlooked. However, many studies found that monoesters not only had hepatotoxicity but also had the characteristics of high detection rates and high levels, which made them potentially toxic to humans when inadvertently ingested [5,18].

There are currently three main hepatotoxicity mechanisms of PAs: (ⅰ) excessive production of reactive oxygen species (ROS) by causing cellular oxidative stress; (ⅱ) promoting apoptosis through the mitochondrial intrinsic pathway; and (ⅲ) induction of liver bile acid stasis. Many studies have provided clues to investigate the toxicity mechanism of PAs, and their results showed that isoline (retronecine-type) and clivorine (otonecine-type) affected the activity of antioxidant enzymes in cells after entering the liver, thereby causing the occurrence of cellular oxidative stress [8,16]. In addition, in the second pathway, the toxicity mechanism of PAs was related to the damage of mitochondria. PAs formed pyrrole-ATP5B adducts in three different kinds of hepatocytes, which reduced intracellular ATP levels and disrupted the homeostasis of mitochondria, thereby triggering mitochondrial dysfunction and cell death [32]. Meanwhile, in the third pathway, the 1,2-unsaturated PAs induced a decrease in the gene expression of various hepatobiliary transporters, enzymes involved in bile acid synthesis and binding and several transcriptional regulators, thereby leading to the destruction of bile acid homeostasis and causing liver injury [33].

Intermedine (Im) is a widely distributed and typical retronecine-type monoester (Figure 1B) that exists in many kinds of plants, such as *Comfrey* [34,35], *Blue heliotrope* [7] and *Asmachilca* [36]. Im N-oxide has also been detected in herbs, such as *Eupatorium perfoliatum* [37] and *Oregano* [38]. Recently, we found that the high level of ImNO in a dark tea sample was 151.33 μg/kg [11]. The previous study demonstrated that *Comfrey* was hepatotoxic to livestock and humans and found Im to be the main PA in this herb [39]. The European Food Safety Agency evaluated the dietary exposure to PAs in European diets in 2015 and found that a variety of PAs were detected in green tea, black tea and chamomile, with high levels of Im in the total PAs [40]. In addition, Im has been detected in the commercially available honey in Australia [10], as well as in the food and animal feed on the Belgian market [41]. In Switzerland, it has been found in plants near streams in many places. After the rainy season, the levels of Im in small streams exceeds the threshold of toxicological concern for genotoxic pollutants in drinking water [5]. Therefore, Im may be one of the PAs exposed to human beings through the food chain and drinking water, and it is necessary to assess its health risk. However, the current lack of toxicological data surrounding Im prevents the systematic evaluation of the health risks of PA compounds. Herein, we evaluated the toxicity of Im to hepatocytes and the potential mechanisms of hepatoxicity in vitro. The cytotoxicity of Im to primary mouse hepatocytes, HepD cells, H_22_ cells and HepG2 cells was evaluated through multiple cytotoxicity evaluation methods. Moreover, Im induced oxidative stress in HepD cells by producing overloaded ROS. The accumulation of ROS with unpaired electrons was particularly reactive to attacking biomacromolecules and accelerated cell death. On the other hand, the destruction of the cell mitochondrial structure and the release of Cyt c demonstrated that Im activated the mitochondria-mediated apoptosis pathway. These results showed that Im induced cytotoxicity by producing excessive ROS and activating the mitochondrial-mediated apoptosis pathway in HepD cells.

## 2. Results

### 2.1. The Cytotoxicity of Eight PAs Compound to Mammalian Cell Lines

**Figure 2:**
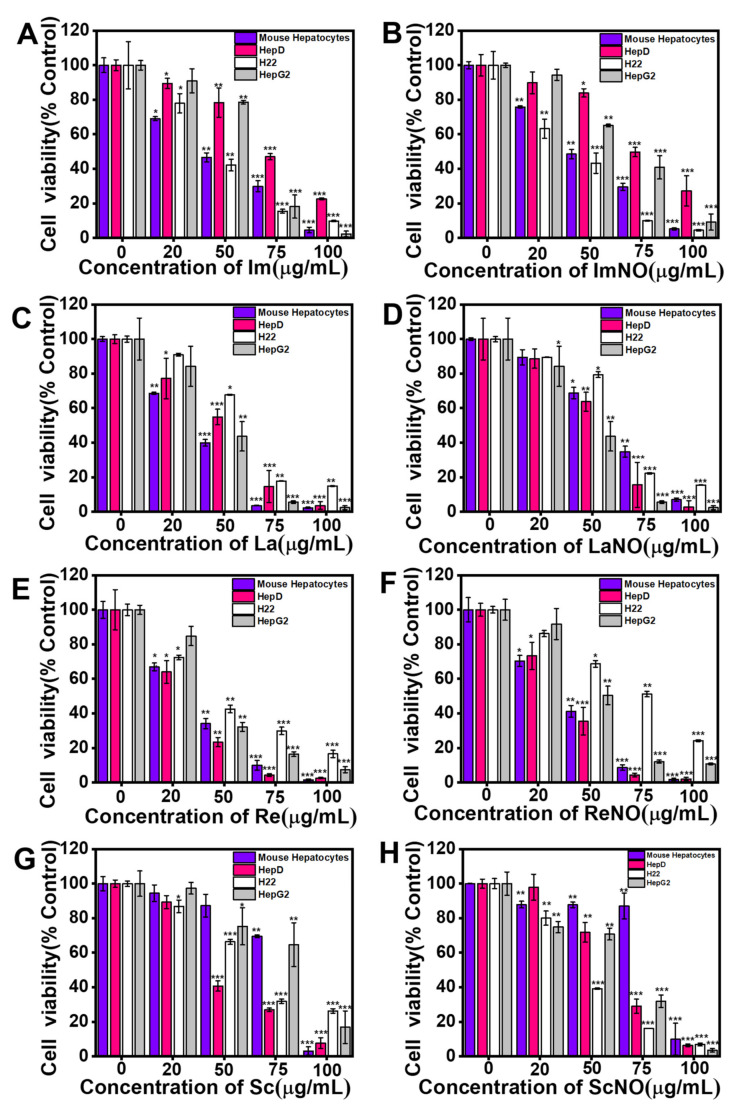
Im, ImNO, La, LaNO, Re, ReNO, Sc and ScNO inhibited the growth of primary mouse hepatocytes, human hepatocytes (HepD), mouse hepatoma-22 (H_22_) and human hepatocellular carcinoma (HepG2) cells. The proliferative ability of primary mouse hepatocytes, HepD cells, H_22_ cells and HepG2 cells was detected using cell counting kit-8 assays after the cells were treated with Im, ImNO, La, LaNO, Re, ReNO, Sc and ScNO. (**A**) The viability of four kinds of cells after treated by Im. (**B**) The viability of four kinds of cells after treated by ImNO. (**C**) The viability of four kinds of cells after treated by La. (**D**) The viability of four kinds of cells after treated by LaNO. (**E**) The viability of four kinds of cells after treated by Re. (**F**) The viability of four kinds of cells after treated by ReNO. (**G**) The viability of four kinds of cells after treated by Sc. (**H**) The viability of four kinds of cells after treated by ScNO. Data represent means ± standard deviation (S.D.) of three independent experiments. *: *p* < 0.05, **: *p* < 0.01 and ***: *p* < 0.001 compared with the control group.

The cytotoxicity of intermedine (Im), intermedine N-oxide (ImNO), lycopsamine (La), lycopsamine N-oxide (LaNO), retrorsine (Re), retrorsine N-oxide (ReNO), senecionine (Sc) and senecionine N-oxide (ScNO) on primary mouse hepatocytes, HepD cells, H_22_ cells and HepG2 cells was firstly investigated by the cell counting kit-8 (CCK-8) assay. After cells were exposed to different concentrations (0, 20, 50, 75 and 100 µg/mL = 0, 67, 167, 250 and 334 µM) of Im, ImNO, La, LaNO, Re, ReNO, Sc and ScNO for 24 h, the cell survival was recorded by CCK-8 and a microplate reader. As shown in Figure 2A–H, the cell survival of the four cells was significantly inhibited by four kinds of PAs and four kinds of PA N-oxides. The results showed that the cell survival rate steadily decreased as the PAs or PA N-oxides concentration increased, indicating that PAs and PA N-oxides had obvious cytotoxicity in different cell lines in a dose-dependent manner (Figure 2A–H).

As shown in Table 1, the half-maximal inhibitory concentration (IC_50_) values of Im to four cells were higher than 100 µM. Compared with the control group, the extremely low concentration of Im initially had a slight inhibitory effect on HepD cells. Afterwards, it had a significant inhibitory effect at 100 μg/mL (334 µM), where only 22% of cells survived (Figure 2A). In addition, it was noteworthy that the IC_50_ values of Im on primary mouse hepatocytes, HepD cells, H_22_ cells and HepG2 cells were 165.13 μM, 239.39 μM, 161.82 μM and 189.11 μM, respectively. It was shown that, in this experiment, Im had the most obvious inhibitory effect on H_22_ cells (Table 1). On the other hand, for HepD cells, the IC_50_ values of Im, La, Re and Sc were 239.39 µM, 164.06 µM, 126.55 µM and 173.71 µM, respectively. Therefore, the order of toxicity in the four alkaloids in this experiment was: Re > La > Sc > Im.

Furthermore, the IC_50_ value of HepD cells exposed to ImNO was 257.98 μM, which was higher than the IC_50_ value of Im at 239.39 μM (Table 1), indicating the toxicity of ImNO to HepD cells to be slightly lower than that of Im. These results were consistent with the previously reported findings that PA N-oxides were lower than the corresponding PAs [17].

### 2.2. Intermedine Inhibited Colony Formation and Migration Ability in HepD Cells

**Figure 3:**
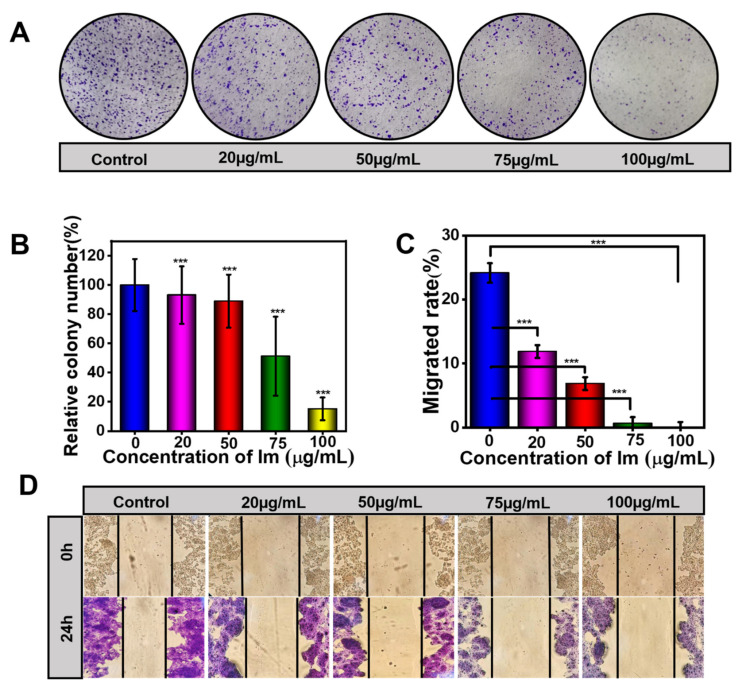
Im inhibited cell colony formation and migration. (**A**)Typical representative photographs of HepD cells that were subjected to a colony formation assay. HepD cells were exposed to various concentrations of Im (0, 20, 50, 75 and 100 µg/mL) for 24 h, followed by staining with crystal violet. (**B**) Quantitative statistics of the number of cells corresponding to A. Negative control: 0 µg/mL. (**C**) Quantitative statistics of the scratch distance of cells corresponding to D. (**D**) For the wound healing assay, HepD cells were treated with 0, 20, 50, 75 and 100 µg/mL Im. Typical representative photographs of HepD cells migration after treatment with Im for 0 h and 24 h. Data represent means ± S.D. of three independent experiments. ***: *p* < 0.001 compared with the control. Negative control: the scratch distance of cells at 0 h.

The above CCK-8 assay has proven that Im had cytotoxicity to HepD cells. To investigate whether Im decreased colony formation, we performed a colony formation analysis on HepD cells [42]. After HepD cells were exposed to different concentrations of Im (0, 20, 50, 75 and 100 µg/mL) for 7 days colony formation decreased significantly with the increase of Im concentrations (Figure 3A). Compared with the control group, the counts of cell cloning formations were only 15.2% with the treatment of Im at 100 μg/mL. This also showed that Im at 75 and 100 μg/mL induced cell death (Figure 3B).

The wound-healing assay was also performed to assess the migration ability of HepD cells after treatment with Im. As shown in Figure 3C,D, the relative distance of cell migration reflected the viability of cells, and Im inhibited the capability of HepD cells in migration. Compared with the initial width, Im at 0, 20, 50, 75 and 100 μg/mL, the relative widths of the wounds were 75.83%, 88.13%, 93.13%, 99.38% and 99.90%, respectively. These data revealed that the migration ability of HepD cells was completely lost when exposed to Im at 100 μg/mL. Taken together, these results showed that Im inhibited colony formation and migration of HepD cells, which further demonstrated the cytotoxic effects of Im on cells.

### 2.3. Intermedine-Induced HepD Cell Apoptosis

**Figure 4:**
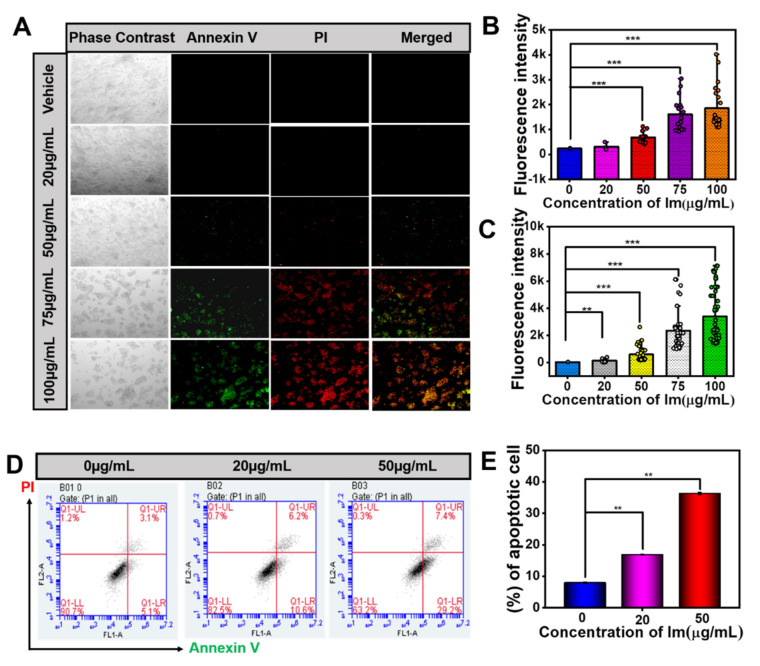
Im-induced HepD cells apoptosis. (**A**) The Annexin V/PI fluorescent images of cells treated with Im (0, 20, 50, 75 and 100 µg/mL) for 24 h (the early apoptotic cells are in green color, and the late apoptotic cells are in green and red color). (**B**) Quantitative statistics of the green fluorescence intensity corresponding to A. (**C**) Quantitative statistics of the red fluorescence intensity corresponding to A. (**D**) HepD cells were exposed to various concentrations of Im (0, 20 and 50 µg/mL) for 24 h, followed by flow cytometry-based apoptosis assay. (**E**) Group data analysis of the percentage of apoptotic cells corresponding to D. Data represent means ± S.D. of three independent experiments. **: *p* < 0.01 and ***: *p* < 0.001 compared with the control.

The results of the above experiment proved the cytotoxicity of Im and its ability to inhibit cell migration. To further verify whether Im induced HepD cells apoptosis, we used Annexin V/propidium iodide (Annexin V/PI) staining assay to detect cell apoptosis. Annexin is a phospholipid-binding protein that selectively binds phosphatidylserine. Phosphatidylserine is mainly distributed inside the cell membrane and is flipped when early apoptosis occurs in cells. The fluorescein isothiocyanate (FITC)-labeled Annexin V binds to phosphatidylserine and detects the occurrence of early-cell apoptosis. Additionally, PI staining detects the occurrence of late-cell apoptosis. The apoptotic populations of HepD cells were detected with Annexin V-FITC/PI staining. As shown in Figure 4A–C, the results showed that the green and red fluorescent signal significantly increased with the growing concentration of Im, indicating that the rate of cell apoptosis increased. Meanwhile, the early and late apoptotic were examined by flow cytometric analysis (Figure 4D,E). The average apoptotic ratios, represented by cells in the Q1- Upper Right (UR) and Q1- Lower Right (LR) quadrants, were 7.93%, 16.85% and 36.29% in HepD cells after exposure to 0, 20 and 50 μg/mL Im. Therefore, Im induced HepD cell apoptosis in a dose-dependent manner. This result was consistent with the results in the cell proliferation test, and suggested that Im reduced the viability of HepD cells by inducing apoptosis.

### 2.4. Intermedine-Triggered ROS Burst

**Figure 5:**
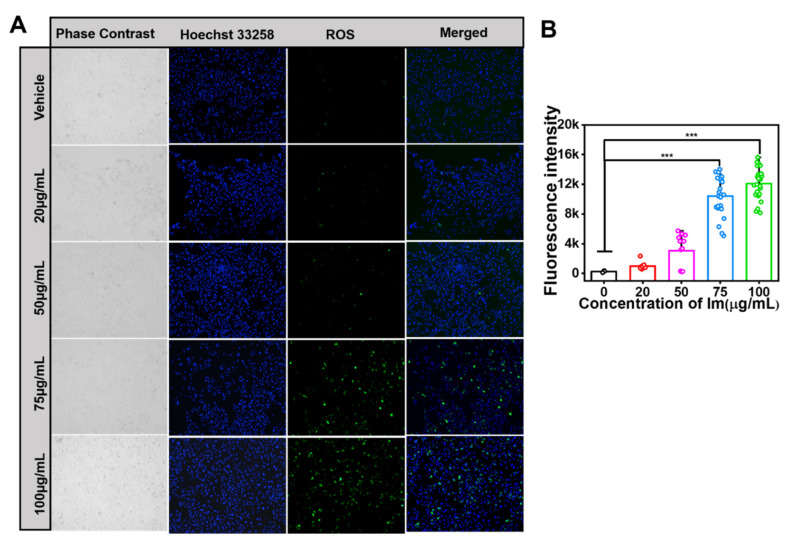
Im-induced ROS production in HepD cells. (**A**) The production of intracellular ROS levels was detected with DCFH-DA (ROS-positive cells are in green) and the nucleus was stained with Hoechst 33258 (nucleus are in blue). HepD cells were incubated with 0, 20, 50, 75 and 100 µg/mL Im for 24 h and the intensity of green fluorescence increased significantly as the concentration of Im increased. (**B**) Quantitative statistics of ROS fluorescence intensity corresponding to A. Data represent means ± S.D. of three independent experiments. ***: *p* < 0.001 compared with the control.

Previous studies reported that PA-induced hepatotoxicity was involved with excessive ROS by cellular oxidative stress. A further experiment was performed to verify Im-induced oxidative stress in HepD cells. The intracellular ROS level was detected with the 2,7-Dichlorodi -hydrofluorescein diacetate (DCFH-DA) probe, and the green fluorescence intensity reflected the level of ROS. The results showed that, after HepD cells were treated with Im, the levels of intracellular ROS increased significantly. As shown in Figure 5A, the levels of ROS increased when HepD cells were treated with 0, 20, 50, 75 and 100 µg/mL Im for 24 h. According to the results of Annexin V-FITC/PI apoptosis assay, when HepD cells were exposed to Im at 0, 20, 50, 75 and 100 µg/mL for 24 h, the apoptosis rates increased significantly, indicating that the Im-induced apoptosis was related to the production of ROS in HepD cells.

### 2.5. Intermedine-Induced Mitochondrial Damage

Apoptotic pathways included both exogenous pathways (death receptor pathway) and endogenous pathways (mitochondrial pathway) [1]. The endogenous pathway is associated with mitochondria, and the morphology of mitochondria is related to its normal function. The drop of the mitochondrial membrane potential is a sign of early apoptosis. When cells receive the apoptosis signal, the permeability of the cell membrane changes and the mitochondrial transmembrane potential is depolarized.

**Figure 6:**
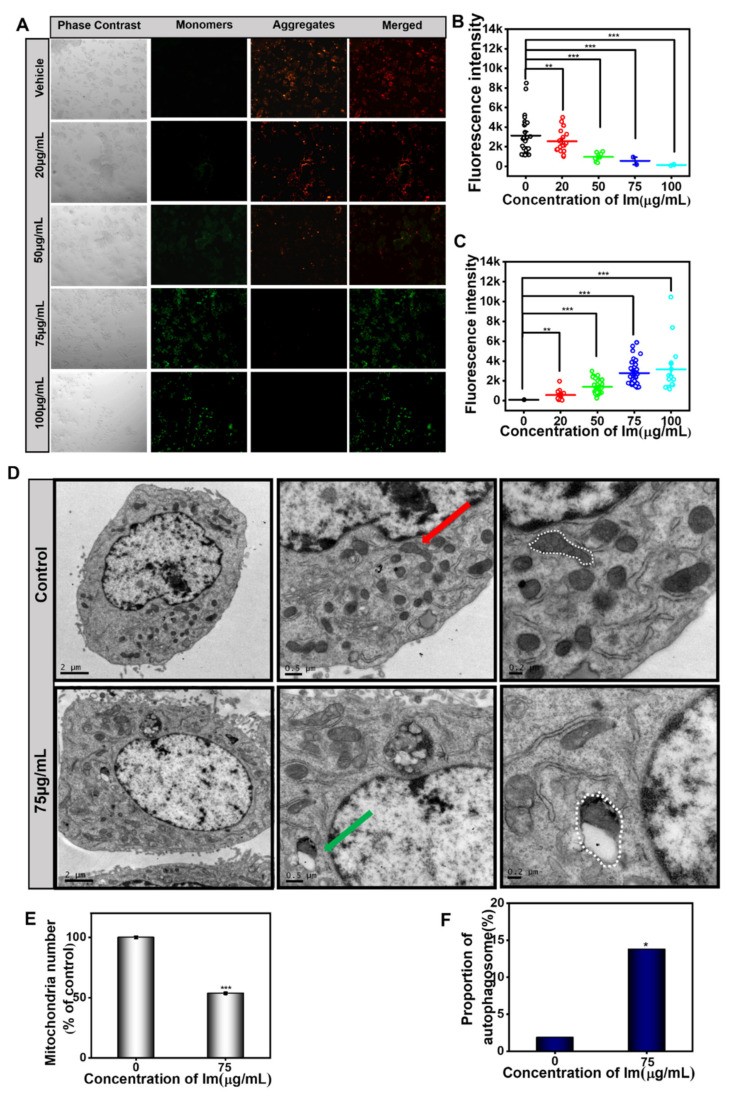
Im-induced mitochondrial damage in HepD cells. (**A**) HepD cells were treated with 0, 20, 50, 75 and 100 µg/mL Im, representative fluorescence images of HepD cells of mitochondrial membrane potential assay. Green fluorescence increased and red fluorescence decreased; Im induced mitochondrial damage and decreased mitochondrial membrane potential. (**B**) Quantitative statistics red fluorescence intensity statistics graph corresponding to A. (**C**) Quantitative statistics green fluorescence intensity statistics graph corresponding to A. (**D**) HepD cells were treated with 0, 75 µg/mL Im and representative images of transmission electron microscope. Red arrows indicated normal mitochondria and green arrows indicated mitochondrial autophagosome. (**E**) The percentage of mitochondria numbers in 0, 75 µg/mL Im-treated HepD cells. (**F**) The percentage of mitochondrial autophagy in 0, 75 µg/mL Im-treated HepD cells. *: *p* < 0.05, **: *p* < 0.01 and ***: *p* < 0.001 compared with the control.

To further verify the mitochondrial damage, the JC-1 fluorescence assay was performed. The results showed that Im induced the loss of mitochondrial membrane potential in HepD cells (Figure 6A). When HepD cells were incubated in Im (0, 20, 50, 75 and 100 µg/mL) for 24 h, the red fluorescence intensity decreased almost 100% and the green fluorescence intensity increased (Figure 6A–C). All results indicated that apoptosis was related to mitochondrial damage and membrane potential loss.

From the TEM images, we found that the nucleus volume of the Im-treated cells increased in size, the cytoplasm shrunk, the mitochondria formed a cup-shaped structure and the isolation membrane expanded into a spherical shape to form autophagosomes (Figure 6D). In contrast, in the control group, mitochondria were mostly slender and had obvious ridges. In addition, compared with the control group, the number of mitochondria decreased significantly in cells treated with 75 µg/mL Im, and a large number of mitochondrial autophagy was observed (Figure 6E,F).

**Figure 7:**
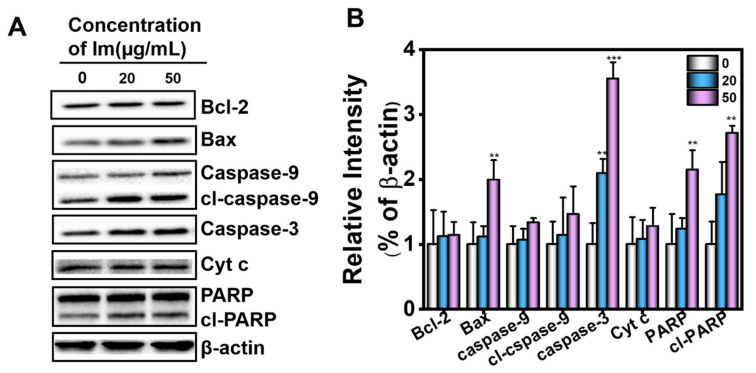
HepD cells were treated with 0, 20 and 50 µg/mL Im. (**A**) Effects of Im on the expression of apoptosis-related proteins B-cell lymphoma-2 (Bcl-2), Bcl-2-Associated X protein (Bax), PARP, cl-PARP, caspase-3, caspase-9, cleaved caspase-9 and Cytochrome c) in HepD cells. HepD cells were treated with 0, 20 and 50 µg/mL Im for 24 h and the expression levels of Bcl-2, Bax, PARP, cl-PARP, caspase-3, caspase-9, cleaved caspase-9 and Cytochrome c (Cyt c) were examined by western blot analysis. (**B**) The statistics graph shows the expression of these proteins corresponding to A. Data represent means ± S.D. of three independent experiments. **: *p* < 0.01 and ***: *p* < 0.001 compared with cells treated with 0 µg/mL Im.

Mitochondrial damage led to changes in mitochondrial membrane permeability, and Cyt c was released from the mitochondria into the cytoplasm. In the cytoplasm, Cyt c, together with caspase-9 and apoptotic protease (Apaf1), formed apoptosome. Subsequently, the apoptosome activated caspase-3 in the cytoplasm to induce hepatocyte apoptosis, and further caused liver injury. To explore the potential apoptotic pathway related to the cellular response to Im, we investigated the activation status of the downstream signaling components of the mitochondrial apoptotic pathway. Exposure of cells to Im led to the activation of several apoptotic signaling molecules, including caspase-3, caspase-9 and their substrate, Poly ADP-ribose polymerase (PARP), in a concentration-dependent manner. As depicted in Figure 7, the Cyt c level in the cytoplasm of HepD cells increased in a dose-dependent manner after 24 h exposure to 0, 20 and 50 µg/mL of Im, indicating that Cyt c was released from the mitochondria into the cytoplasm. Caspase-9 is the initiator of the apoptosis program, and caspase-3 is the executor of apoptotic cell death. The up-regulation of caspase-3, caspase-9 and cleaved caspase-9 (cl-caspase-9) expression levels indicated that the caspase-dependent apoptotic pathway was involved in the Im-induced apoptosis of HepD cells. In addition, the intensity of the cleaved PARP (cl-PARP) fragment was directly proportional to the concentration of Im, and the levels of caspase-3 and cl-PARP significantly increased at 20 and 50 µg/mL (Figure 7). PARP is the cleavage substrate for caspase-3 and is activated to cl-PARP; the appearance of cl-PARP marks the activation of caspase-3 and the happening of cell apoptosis. In summary, these data clearly indicated that Im induced apoptosis by disrupting mitochondrial homeostasis, inducing the loss of mitochondrial membrane potential and activating the release of Cyt c from mitochondria to the cytoplasm in HepD cells.

## 3. Discussion

Im is present in numerous plants and plant extracts and is a PA commonly found in *Comfrey* [35]. Previous research has investigated the toxicity of different PAs and shown that Im was less toxic and had a lower carcinogenic risk [43]. However, in our study, we found that Im was actually toxic to mammalian cell lines. We speculated that the reason for the difference in toxicity was that the concentrations of Im in our study were 0, 20, 50, 75 and 100 µg/mL (0, 67, 167, 250 and 334 µM), which were higher than the concentrations of Im in other experiments [44]. Higher concentrations induced more severe cytotoxicity. PAs were experimented in different species and cell lines, treated with different incubation times, doses or concentrations and different endpoints or methods [43], which led to different experimental results. These could be the reasons for the differences in the cytotoxicity of PAs. In this study, the change of cell morphology was observed under an ordinary optical microscope (Figure S1). On the other hand, we found Im caused a burst of ROS in HepD cells (Figure 5A), making the mitochondrial membrane potential decrease (Figure 6A). Moreover, Im destroyed the mitochondrial structure, making the mitochondrial morphology change, and mitochondrial autophagosomes appeared in cells (Figure 6D). Autophagy is an evolutionarily conserved and genetically programmed lysosomal degradation pathway that digests some cytoplasmic components to maintain intracellular homeostasis and is used to cope with various metabolic stresses [45,46]. Cellular necrosis is a passive death due to pathology. When cells are damaged by external sources, the membrane permeability of necrotic cells increases, resulting in the swelling of cells and deformation or enlargement of organelles. Mitochondria are intracellular energy reservoirs that are critical to cell survival. Mitochondrial autophagy is a specific form of autophagy used to remove damaged mitochondria to maintain intracellular environmental homeostasis and normal function [47]. Cell death can be triggered by unrestricted autophagy occurring in mitochondria, as well as by the damage of organelles [48]. Autophagy and apoptosis are two interlinked modalities by which cells control apoptosis by regulating the autophagic process. At the same time, the activation of caspase enzymes during apoptosis could, in turn, control the increase or decrease of autophagy. Many studies have shown that autophagy promotes apoptosis and could be a mechanism of cell death, degrading cellular components and thus indirectly activating the apoptotic mechanism of cells [49,50]. In this study, the burst of ROS triggered by Im disrupted the balance of mitochondrial homeostasis and induced apoptosis. The expression of proteins related to apoptosis significantly increased (Figure 7). These results indicated that Im entered the cells and caused damage to them.

Compared with other PAs in diester structures, PAs with monoester structures could not generate more serious toxicity, but PAs with low toxicity could produce severe cytotoxicity when combined with other PAs [35]. Therefore, the cytotoxicity of Im should not be neglected. Herein, we confirmed that the toxicity of Im to cell lines was derived from mammalian tissues, including primary mouse hepatocytes, HepD cells, H_22_ cells and HepG2 cells. In vitro, we found that Im significantly caused apoptosis in HepD cells and induced cytotoxicity in a dose-dependent manner. As depicted in Figure 2A, cell mortality became more pronounced when the concentration of Im increased to 100 μg/mL. For the four mammalian cells, the most pronounced inhibitory effect of Im was observed on H_22_ cells (Table 1), which probably involved the different toxic responses of Im to different species [51,52]. The previous study reported that the differences in esterase activity were responsible for the differences in the metabolism of clivorine (otonecine-type PAs), which was observed in normal human liver L-02 cells [53,54]. Thus, we speculated that the esterase activity of Im was similar to that of clivorine and that Im was strain-related. Therefore, the different mortality rates of human and rat cells indicated that the different toxic responses of Im to mammalian cells possibly resulted from the different esterase activities. Moreover, the results of the colony formation experiment showed that the number of colony formations in Im-treated groups decreased in a concentration-dependent manner (Figure 3A), indicating Im had cytotoxicity to HepD cells.

On the other hand, to assess whether Im inhibited the capability of HepD cells in migration, the cell wound-healing assay was prepared. The wound-healing assay is a common method to evaluate the ability of cells to migrate and invade [55,56]. When cells are disturbed by harmful substances, their ability to move is affected. The wound-healing assay proved that Im severely affected the mobility of HepD cells. Collectively, the results of in vitro experiments clearly demonstrated that Im not only markedly inhibited the proliferation, clone formation and migration ability of HepD cells but also produced toxicity and induced apoptosis of HepD cells.

To observe the apoptotic cells, Annexin V/PI dyes were used to label HepD cells, which were treated with Im at different concentrations (0, 20, 50, 75 and 100 μg/mL) for 24 h. As observed by Annexin V/PI apoptosis assay, cell viability was not affected in the control group without any treatment. In contrast, several HepD cells were killed with the treatment of Im, as indicated by the intense green and red fluorescent signals (Figure 4A–C). The number of apoptotic cells was further assessed by flow cytometric analysis (Figure 4D). HepD cells were incubated with three different concentrations of Im (0, 20 and 50 μg/mL). As shown in Figure 4E, higher levels of cell death were detected after incubation with Im compared with the control group. These data further demonstrated that Im significantly caused cell apoptosis.

Numerous studies have found that hepatotoxicity caused by PAs is attributed to hepatocyte apoptosis [13,53,57]. The main ways in which PAs caused hepatotoxicity increased intracellular ROS and mitochondria-mediated apoptosis [1]. To verify whether ROS was involved in Im-induced cell apoptosis, the ROS generation ability of HepD cells was tested using DCFH as a ROS indicator (Figure 5A). In our study, we found that the concentration of 75 µg/mL and 100 µg/mL Im resulted in significant increases in ROS levels. The green fluorescence showed a similar increasing trend as the concentration of Im increased. The fluorescence intensity statistics graph intuitively reflected the increase in fluorescence intensity (Figure 5B), and the results showed that ROS was involved in Im-induced cell apoptosis.

However, the molecular mechanism of Im-induced ROS production remains unknown. We speculated that the mechanism was similar to that of isoline-induced oxidative damage in mouse cells. Mitochondria are the place in cells where large amounts of ATP are produced [16,58]. The process is accompanied by the generation of a large number of ROS. Simultaneously, the glutathione and antioxidant enzymes in cells, such as catalase (CAT), glutathione peroxidase (GPX), and glutathione s-transferase (GST), immediately eliminate ROS to maintain homeostasis [16,59]. However, when excessive endogenous ROS production could not be effectively removed by intracellular antioxidants, intracellular oxidative stress was triggered [53]. Excessive ROS with an unpaired electron in intracellular attack macromolecules (such as lipids, proteins and DNA) thereby causing cellular damage. Therefore, we hypothesized that Im induced intracellular oxidative stress and generated excessive ROS, which induced cell apoptosis.

The present study, for the first time, directly demonstrated that Im induced hepatotoxicity through mitochondria-mediated apoptosis. Mitochondrial membrane potential loss is an early marker event of the apoptosis pathway [13]. This research uncovered that Im significantly induced the loss of mitochondrial membrane potential (Figure 6A). We used TEM to observe the morphology of Im-treated HepD cells. From the mitochondria images, we found that the mitochondria formed a cup-shaped structure, and the isolated membrane expanded into a spherical shape to form autophagosomes (Figure 6D). We also found that the number of mitochondria decreased in HepD cells treated with 75 µg/mL Im. Mitochondrial autophagy is one of the pathways of catabolic dysfunction or selective autophagy of excess mitochondria [60]. The presence of mitochondrial autophagy indicated that the clearance mechanism was activated and the homeostasis of mitochondria was disrupted. Further studies revealed that Im-induced hepatotoxicity was related to the release of Cyt c and the activation of caspase-3, caspase-9 and PARP in cells. The caspase family of proteins are involved in all apoptotic pathways [61,62], as evidenced by the increased expression of Cyt c in the cytoplasm and the dramatically activated caspase-3, caspase-9 and cl-PARP. In conclusion, our results showed that Im induced mitochondria-mediated apoptosis by inducing mitochondria autophagy, loss of mitochondrial membrane potential and Cyt c release, as well as activation of caspase-3 and caspase-9.

On the other hand, in cell proliferation assays, we found that the toxicity of monoester Im was much lower than the other three kinds of PAs in HepD cells. According to previous reports, the metabolic activation of monoesters in human liver microsomes was different from that of diesters in the absence of glutathione (GSH) conjugates and DNA adducts [28]. Therefore, we deduced that the lower degree of binding between Im and biomacromolecules could account for the lower toxicity of Im. In addition, we also found that both PAs and PA N-oxides had an inhibitory effect on cell viability, while ImNO was less toxic than Im. We speculated that this phenomenon was related to the different metabolic activations of Im and ImNO in cells. Previous studies have demonstrated that the hepatotoxicity of PA N-oxides was similar to that of PAs, but its toxic potency was much lower than that of the corresponding PAs [6,17]. Because the low lipophilicity of PA N-oxides resulted in poor absorption of PA N-oxides by cells, PA N-oxides were less toxic than PAs. It was thus extrapolated that the low lipophilicity of ImNO resulted in the lower hepatotoxicity of ImNO in HepD cells, which required further experiments to verify.

## 4. Conclusions

In conclusion, we demonstrated the toxicity of Im on different hepatocytes, and the cytotoxicity was directly related to the burst of ROS. Overloaded ROS with unpaired electrons were overactive and attacked the biological macromolecules, causing damage to the organelle structure and ultimately resulting in cell death. Furthermore, the mechanism of Im-induced apoptosis was related to the mitochondrial-mediated apoptotic pathway. The mitochondrial membrane permeability increased and Cyt c in the mitochondria was released into the cytoplasm, which activated caspase-9. The caspase-9 then initiated the caspase-3 apoptotic pathway and caused cell death. Considering the high detection rate and high levels of Im, it is necessary to conduct more research to provide evidence for limiting the levels of Im in medicinal herbs and foods.

## 5. Materials and Methods

### 5.1. Chemicals and Reagents

Intermedine (Im, Purity: 99%), intermedine N-oxide (ImNO, Purity: 96%), lycopsamine (La, Purity: 98%), lycopsamine N-oxide (LaNO, Purity: 93%), retrorsine (Re, Purity: 99%), retrorsine N-oxide (ReNO, Purity: 96%), senecionine (Sc, Purity: 99%) and senecionine N-oxide (ScNO, Purity: 91%) were purchased from Shanghai standards Biotechnology Co., Ltd. (Shanghai, China). The Cell Counting Kit-8 (CCK-8) reagent was acquired from Dojindo (Kumamoto, Japan). Dimethyl-sulfoxide (DMSO) and Collagenase IV were obtained from Sigma Aldrich Chemical Co. (St. Louis, MO, USA). Roswell park memorial institute (RPMI) 1640 medium, Minimum essential medium (MEM), Dulbecco’s modified Eagle’s medium (DMEM) and 1×phosphate-buffered saline (PBS, pH 7.2–7.4) were obtained from HyClone (Waltham, MA, USA). Trypsin-ethylene diamine tetraacetic acid (EDTA), penicillin-streptomycin and fetal bovine serum (FBS) were purchased from Gibco (Waltham, MA, USA). The reactive oxygen species assay kit, Hoechst 33258, Annexin V-FITC apoptosis detection kit, mitochondrial membrane potential assay kit with JC-1, enhanced butyleyanoacrylate (BCA) protein assay kit, sodium dodecyl sulfate-polyacrylamide gel electrophoresis (SDS-PAGE) sample loading buffer 5×, crystal violet staining solution and radio immunoprecipitation assay (RIPA) Lysis Solution were all bought from Shanghai beyotime Biotechnology Co., Ltd. (Shanghai, China). The mitochondrial protein extraction kit was obtained from Shanghai X-Y Biotechnology Co., Ltd. (Shanghai, China). Institute of Cancer Research (ICR) mice (female, 20–30 g) used in the experiment were purchased from Shanghai Jihui Experimental Animal Breeding Co., Ltd. (Shanghai, China). The animal experiments complied with the Animal Research: Reporting in Vivo Experiments (ARRIVE) guidelines and were approved by the Institutional Animal Care and Ethics Committee (Approval No. SCXK2014-0004, 2014/09).

### 5.2. Cell Culture and Reagents

**Figure 8:**
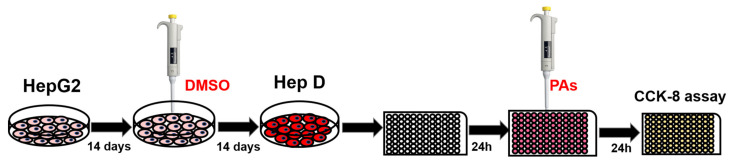
Cultivation and incubation scheme for HepD cells. HepG2 cells were grown in MEM medium supplemented with 10% (*v*/*v*) fetal bovine serum, 100 U/mL penicillin and 100 U/mL streptomycin. For HepG2 cell differentiation, a two-step procedure was used to prepare cell proliferation assays by first culturing HepG2 cells in MEM medium for 14 days followed by further culturing in medium with 1.7% DMSO for 14 days and then differentiating HepG2 cells into HepD cells.

Mouse hepatoma-22 cells (H_22_) and human hepatocellular carcinoma (HepG2) were purchased from Shanghai X-Y Biotechnology Co., Ltd. (Shanghai, China). Primary mouse hepatocytes were separated and extracted according to previous methods [63]. Human hepatocytes (HepD) were obtained by incubating, according to the previous study [64], and HepG2 cells were cultured in MEM medium supplemented with 10% FBS and 1% antibiotics (100 U/mL penicillin and 100 U/mL streptomycin) in presence of 1.7% DMSO. After seeding, HepG2 cells actively divided once they reached confluence and incubated for 14 days without DMSO, then a further 14 days for differentiation. The cells became smaller and triangular, spindle-shaped or polygonal in shape. In addition, the nucleus became smaller, the nucleolus reduced and the secretion of intracytoplasmic granules increased. The cytoplasm was lined with vacuoles and the morphology evolved into that of normal human hepatocytes [64]. The process of incubating HepG2 cells into HepD cells was shown in Figure 8. H_22_ cells, HepG2 cells and primary mouse hepatocytes were cultured in RPMI 1640, MEM medium and Dulbecco’s modified eagle medium (DMEM) medium supplemented with 10% FBS and 1% antibiotics, respectively. Four kinds of cells were routinely cultured at 37 °C in a humidified incubator with an atmosphere of 5% CO_2_. Primary antibodies, including the Bcl-2, Bax, caspase-3, caspase-9, cleaved caspase-9, PARP, cleaved PARP and anti-β-actin antibody were purchased from Cell Signaling Technology (Danvers, MA, USA). The primary antibody for cytochrome c (Cyt c) was purchased from Abcam (Cambridge, MA, USA).

### 5.3. Preparation of Primary Mouse Hepatocytes

Six-week-old female ICR mice (20–30g weight) were kept under standard laboratory conditions and provided access to commercial diet and water. The cell experiments were carried out in a sterile environment. Primary mouse hepatocytes were prepared as follows. First, mice were sacrificed by severing the neck, and the mouse liver was taken out carefully and washed in sterile PBS buffer three times. Next, the liver adipose tissue was removed with forceps. Subsequently, the sample was placed in a sterile dish filled with PBS buffer. After that, the sample was cut into pieces with surgical scissors and filtered by a 100-mesh filter. The filtrate was transferred to a 15 mL centrifuge tube and centrifuged at 167× *g* for 3 min. The supernatant was discarded and a volume of 5 mL of collagenase IV (10 mg/mL) was added to the sample. Then, the sample was enzymatically digested at 37 °C for 10 min and the tube was mixed gently once every 2 min. After digestion, the liver tissue was centrifuged at 167× *g* for 3 min. The pellet was resuspended in PBS buffer at 4 °C, centrifuged at 167× *g* for 3 min and the supernatant was discarded. High-purity mouse hepatocytes were obtained by resuspending the pellet in PBS buffer 3 times.

The freshly extracted high purity hepatocytes were seeded in DMEM medium containing 10% FBS and 1% penicillin-streptomycin, The medium was renewed after 72 h and changed every 24 h.

### 5.4. Cell Viability Assay

The CCK-8 assay was used to quantify the cytotoxicity of pyrrolizidine alkaloids to cells [13]. CCK-8 allows for particularly convenient assays by utilizing a highly water-soluble tetrazolium salt. WST-8 is a water-soluble tetrazolium salt that produces a water-soluble formazan dye upon reduction in the presence of an electron mediator. In the presence of electronic coupling reagents, WST-8 is reduced by dehydrogenase in the mitochondria to produce an orange–yellow formazan dye that is proportional to the number of living cells. Through colorimetry, the number of living cells is dynamically quantified.

Cells were seeded in a 96-well culture plate at a density of 2 × 10^4^ cells per well in 100 µL medium for 24 h. After cell attachment, cells were treated with serial concentrations of Im, ImNO, La, LaNO, Re, ReNO, Sc and ScNO at 0, 20, 50, 75 and 100 µg/mL. After incubation at 37 °C for 24 h, the original medium was aspirated and cells were washed with PBS solutions; then, a volume of 100 µL of medium contained with 10% CCK-8 was added to each well and the 96-well microplate was incubated at 37 °C for 30 min. Finally, the absorbance was measured with a multimode microplate reader (Thermo Scientific, Berlin, Germany) at 450 nm. The cell viability of the control group was deemed as 100%, and the cell viability of other groups was calculated with the following formula:

(*OD _sample_* − *OD _blank_*/*OD _control_* − *OD _blank_*) × 100% (1)

### 5.5. Cell Clone Formation Assay

HepD cells were seeded in a 6-well plate at a density of 500 cells/well and cultured in MEM-1.7% DMSO medium. Cells were incubated for 7 days and supplemented with 0, 20, 50, 75 and 100 µg/mL of Im, and then the medium was discarded. Next, cells were fixed with 4% paraformaldehyde for 10 min at room temperature. After being rinsed thrice with PBS, cells were stained with 0.1% crystal violet solution for 30 min. Finally, the number of cell clones were counted and photographed with a light microscope (Olympus Corporation, Tokyo, Japan; magnification, ×40).

### 5.6. Detection of Apoptosis

HepD cells were seeded in a laser confocal glass-bottom culture dish with a density of 2 × 10^5^ cells/mL. After incubation at 37 °C and 5% CO_2_ for 24 h, cells were treated with serial concentrations of Im at 0, 20, 50, 75 and 100 µg/mL. The control group was treated with PBS. According to the instructions of the Annexin V/PI cell apoptosis detection kit, 200 μL PBS buffer, 5 μL Annexin-V FITC green fluorescent dye and 0.4 μL PI red fluorescent dye were added to a 1.5 mL centrifuge tube. Then, cells were observed, and the fluorescence photos were obtained with a fluorescence microscope (Olympus Corporation, Tokyo, Japan).

The apoptosis assessment was detected by flow cytometry using Annexin V/PI dye assay. After treatment with Im for 24 h, both floating and non-floating cells were harvested and washed with cold PBS, and then cells were resuspended in 200 μL PBS buffer containing 5 μL Annexin-V FITC green fluorescent dye and 0.4 μL PI red fluorescent dye. They were then incubated at 37 °C for 30 min in the dark. The number of the apoptotic cells was quantified by flow cytometry (BD Biosciences, Franklin Lakes, NJ, USA) and analyzed by C6 software (BD Biosciences, Franklin Lakes, NJ, USA).

### 5.7. The Cell Wound Healing Assay

HepD cells were seeded in a 6-well plate at a density of 1 × 10^6^ cells/well. When the cell confluence reached approximately 90%, a 200 µL pipette was used to scratch the wells. Cells were then washed with PBS twice and cultured with MEM-1.7%DMSO medium. The wound healing was imaged using a light microscope at 0 and 24 h (Olympus Corporation, Tokyo, Japan; magnification, ×20).

### 5.8. Cellular Oxidative Stress Induced Apoptosis

ROS are common products of normal cellular metabolism, which is mainly produced in mitochondria. When the rate of eliminating oxidative free radicals is equal to the rate of ROS production, cells maintain the normal metabolism. However, when foreign substances disrupt this balance, it leads to excessive production of ROS, which attacks DNA, RNA, proteins and lipids, causing cellular damage. Therefore, detection of the amount of ROS in cells could be closely related to the survival status of cells. HepD cells were seeded at a density of 2 × 10^5^ in a laser confocal glass-bottom culture dish at 1.5 mL per well. After 24 h, cells were treated with Im at final concentrations of 0, 20, 50, 75 and 100 µg/mL and the ROS fluorescent stain was prepared, a 1.5 mL centrifuge tube was added with 200 µL PBS buffer, 5 µL DCFH-DA reactive oxygen stain and 0.2 µL Hoechst 33258 nuclear blue fluorescent dye. The original medium in the dish was removed, and the ROS fluorescent stain was added before cells were incubated at 37 °C for 30 min, protected from light. Finally, cell apoptosis was observed and the fluorescent photos were taken by the fluorescence microscope (Olympus Corporation, Tokyo, Japan).

### 5.9. Mitochondrial Membrane Potential

The logarithmic phase cells were digested from the culture dish and added to the laser confocal glass-bottom culture dish, and 1.5 mL of cell suspension was added to one dish at a density of 3 × 10^5^. After incubation for 24 h, HepD cells were exposed to Im at concentrations of 0, 20, 50, 75 and 100 μg/mL for another 24 h. According to the mitochondrial membrane potential detection kit manual, cells were mixed with 1 mL JC-1 staining buffer (1×) in each small dish for 30 min. Then, the culture medium was aspirated and cells were gently washed with PBS twice. Next, the fluorescent images were acquired using the fluorescence microscope (Olympus Corporation, Tokyo, Japan).

### 5.10. Cell Morphological Observation

Transmission electron microcopy (TEM) was employed to observe the morphology of apoptotic cells. HepD cells were seeded in a 6-well plate at a density of 2 × 10^5^ cells/well and cultured in a cell incubator for 24 h. HepD cells were then treated with Im at final concentrations of 0, 75 μg/mL for 24 h. After that, HepD cells were washed twice with PBS and harvested by centrifuging at 167× *g*. The supernatant was removed and 1 mL 2.5% glutaraldehyde was added to the centrifuge tube, placed at 4 °C for more than 4 h. After fixation overnight, the cells were washed with PBS buffer three times. Then, 2% osmium acid was added to the pellet and fixed for 4 h, and cells were rinsed with PBS. Next, cells were dehydrated using gradients of 30%, 50%, 70%, 90%, 95% and 100% absolute ethanol containing anhydrous copper sulfate for 10 min each time. Finally, cells were embedded in Spurr resin, embedded and polymerized at 70 °C for 24 h and an ultrathin cell transmission electron microscope section was made. The morphology of cells and mitochondrial was observed by TEM.

### 5.11. Cell Mitochondrial Protein Extraction and Western Blotting

Cellular mitochondrial protein extraction was used with the mitochondrial protein extraction kit. Briefly, HepD cells (2 × 10^7^ cells/well) were cultured in a 6-well plate for 24 h. Then, the cells were exposed to Im at concentrations of 0, 20, 50, 75 and 100 µg/mL for 24 h.

According to the instructions of the mitochondrial protein extraction kit, 200 µL RIPA lysis buffer was added to each well and cells were collected in a 1.5 mL centrifuge tube with a cell scraper, then centrifuged 1000× *g* for 5 min at 4 °C. The supernatant was discarded and the operation was repeated twice. Then, 500 µL extraction reagent A was added to the cell pellet and, after standing for 5 min, the cell suspension was sucked into a homogenizer and ground up and down 20 times. Next, the slurry was centrifuged at 4 °C and 1000× *g* for 5 min. The supernatant was aspirated into another 1.5 mL centrifuge tube and, after being centrifugated at 1000× *g* for 10 min at 4 °C, the supernatant was collected and the precipitate was discarded. The supernatant was centrifuged at 4 °C and 10000× *g* for 20 min. The purpose of gradient centrifugation was to separate the mitochondria. The 200 µL pre-cooled extraction reagent B was then added to the precipitate, which was collected by centrifugation again at 10,000× *g*, 4 °C for 20 min. Finally, the 100 µL extraction reagent C was added to the precipitate and placed at 4 °C for 30 min, followed by centrifugation at 10,000× *g* for 10 min at 4 °C, and the supernatant was collected as cellular mitochondrial protein.

HepD cells were lysed with RIPA buffer to obtain protein. Protein concentrations were quantified with the BCA protein assay kit. Proteins were separated by 10% SDS-PAGE of 20 µg/well and transferred to polyvinylidene difluoride membranes. The membranes were blocked with 5% nonfat milk at room temperature for 2 h and incubated with the appropriate primary antibodies overnight at 4 °C. The membranes were washed 3 times with TBST and incubated with the peroxidase-conjugated secondary antibodies for 1 h at 37 °C. The protein bands were visualized using the horseradish peroxidase (HRP) reaction with its substrates via ECL chemiluminescence.

### 5.12. Statistical Analysis

All data represent the mean ± standard deviation (S.D). of three independent experiments. The significance of differences was determined using the one-way analysis of variance (ANOVA). Differences were considered significant if *p* < 0.05 (*) and highly significant when *p* < 0.01 (**) or *p* < 0.001 (***). Image J was employed to analyze the western blot band intensity and the fluorescence intensity, and OriginPro 2018 software (OriginLab, Northampton, MA, USA) was used for statistical analysis.

## Figures and Tables

**Table 1:** IC_50_ values (µM) of intermedine (Im), intermedine N-oxide (ImNO), lycopsamine (La), lycopsamine N-oxide (LaNO), retrorsine (Re), retrorsine N-oxide (ReNO), senecionine (Sc) and senecionine N-oxide (ScNO) in primary mouse hepatocytes, HepD cells, H_22_ cells and HepG2 cells.

	Cells	Hepatocytes	HepD	H_22_	HepG2
PA					
Im		165.13 ± 15.70	239.39 ± 14.83	161.82 ± 21.91	189.11 ± 6.11
ImNO		170.61 ± 3.91	257.98 ± 20.47	143.79 ± 14.50	206.45 ± 7.31
La		140.28 ± 1.77	164.06 ± 15.53 **	192.72 ± 6.68	155.10 ± 14.86 *
LaNO		198.43 ± 30.23 *	178.79 ± 17.47	204.58 ± 5.51 *	181.76 ± 8.52
Re		138.68 ± 11.60	126.55 ± 7.98 ***	173.41 ± 4.88	157.85 ± 12.86
ReNO		146.26 ± 12.69	140.81 ± 7.58 **	238.48 ± 28.26 **	173.05 ± 4.31
Sc		245.52 ± 9.79 **	173.71 ± 6.88 *	215.13 ± 2.26 *	253.94 ± 20.51 **
ScNO		280.86 ± 12.83 ***	199.97 ± 9.02	158.38 ± 2.91	186.81 ± 6.68

Data are expressed as means ± S.D. of triplicate experiments performed independently. *: *p* < 0.05, **: *p* < 0.01 and ***: *p* < 0.001 compared with the IC_50_ values of cells treated with Im.

## Data Availability

Data is contained within the article.

## Supplementary Materials

The following are available online at https://www.mdpi.com/article/10.3390/toxins13120849/s1, Figure S1: The morphology changes of primary mouse hepatocytes, human hepatocytes (HepD), mouse hepatoma-22 (H22) and human hepatocellular carcinoma (HepG2) cells treated with different concentrations (0, 20, 50, 75, 100 µg/mL) of Intermedine (Im).

## Abbreviation

PAs	Pyrrolizidine alkaloids
HSOS	Hepatic sinusoidal obstruction syndrome
Im	Intermedine
ImNO	Intermedine N-oxide
La	Lycopsamine
LaNO	Lycopsamine N-oxide
Re	Retrorsine
ReNO	Retrorsine N-oxide
Sc	Senecionine
ScNO	Senecionine N-oxide
HepG2	Human hepatocellular carcinoma
HepD	HepG2 cells that were cultured in 1.7% DMSO
H_22_	Mouse hepatoma-22 cells
ROS	Reactive oxygen species
Cyt c	Cytochrome c
