# Supplementary material for: Hepatotoxicity of Pyrrolizidine Alkaloid Compound Intermedine: Comparison with Other Pyrrolizidine Alkaloids and Its Toxicological Mechanism
Source: Toxins (Basel). 2021 Nov 28;13(12):849. doi: 10.3390/toxins13120849 (PMC8709407; doi:10.3390/toxins13120849)
Supplement: Supplementary file 1 [file toxins-13-00849-s001.zip › toxins-1470105-supplementary.pdf]

toxins

Supplementary Material

Hepatotoxicity of pyrrolizidine alkaloid compound intermedine: comparison with other pyrrolizidine alkaloids and its toxicological mechanism

Ziqi Wang [1,2], Haolei Han [2,3], Chen Wang [2,4], Qinqin Zheng [2,3], Hongping Chen [2,4], Xiangchun Zhang [2,4,*] and Ruyan Hou [1,*]

1 State Key Laboratory of Tea Plant Biology and Utilization, School of Tea and Food Science & Technology, Anhui Agricultural University, Hefei 230036, China; wangziqi199710@163.com
2 Tea Research Institute, Chinese Academy of Agricultural Sciences, Hangzhou 310008, China; 18482004388@163.com (H.H.); wangchen@tricaas.com (C.W.); zhengqinqin@tricaas.com (Q.Z.), thean27@tricaas.com (H.C.)
3 Graduate School, Chinese Academy of Agricultural Sciences, Beijing 100081, China
4 Key Laboratory of Tea Quality and Safety & Risk Assessment, Ministry of Agriculture, Hangzhou 310008, China
* Correspondence: zhangxc@tricaas.com (X.Z.); hry@ahau.edu.cn (R.H.)

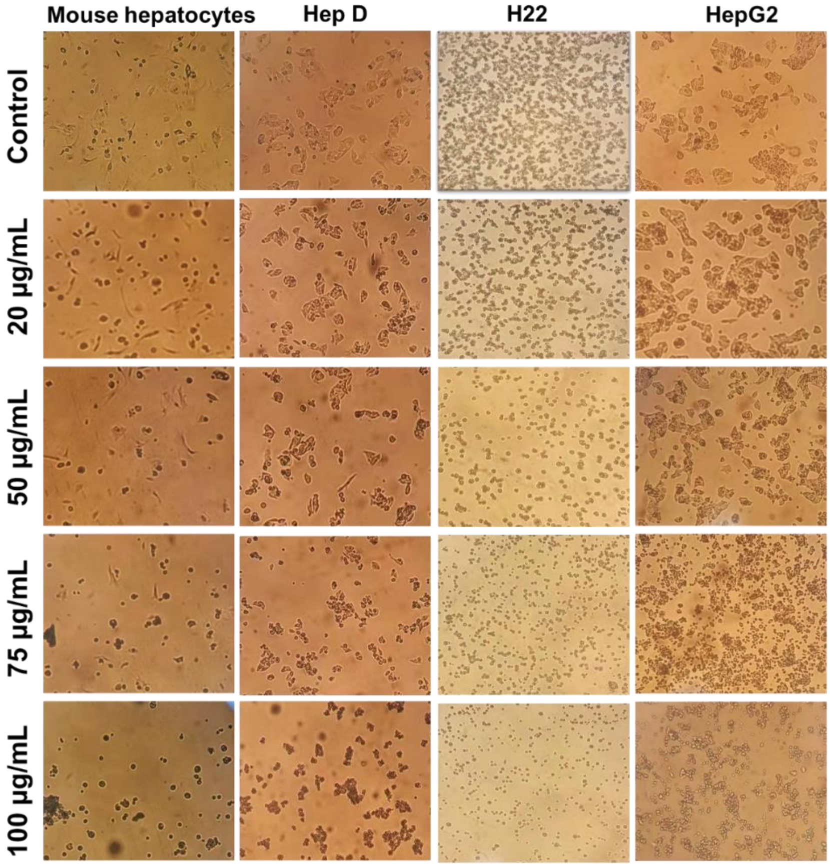

Figure S1. The morphology changes of primary mouse hepatocytes, human hepatocytes (HepD), mouse hepatoma-22 (H_{22}) and human hepatocellular carcinoma (HepG2) cells treated with different concentrations (0, 20, 50, 75, 100 μg/mL) of Intermedine (Im).
